# Supplementary figures and images for: A human centered design approach to define and measure documentation quality using an EHR virtual simulation
Source: PLoS One. 2024 Aug 19;19(8):e0308992. doi: 10.1371/journal.pone.0308992 (PMC11332943; doi:10.1371/journal.pone.0308992)

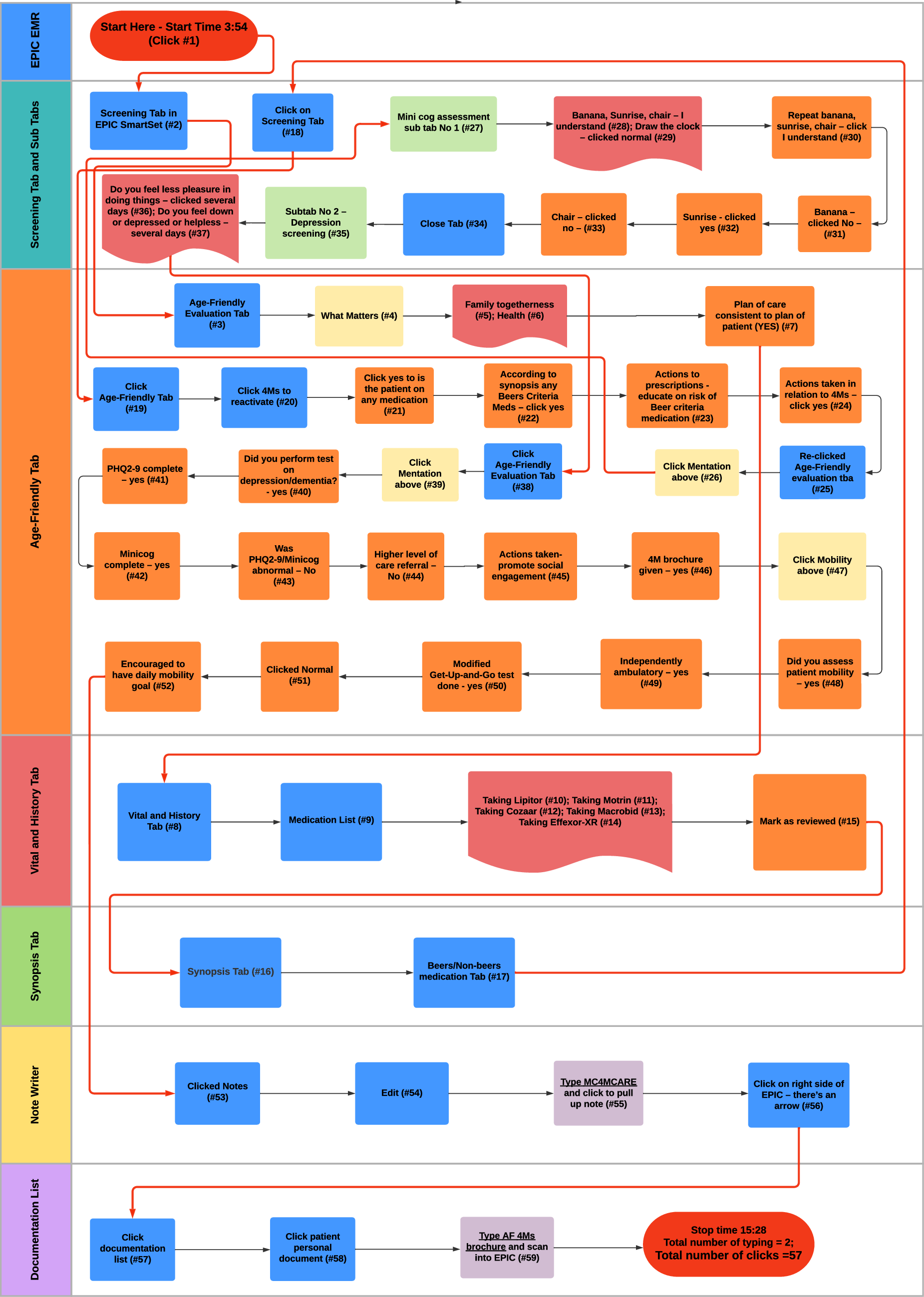

Supplement: S1 Fig — (TIF) [file pone.0308992.s001.tif]
