# Supplementary material for: A human centered design approach to define and measure documentation quality using an EHR virtual simulation
Source: PLoS One. 2024 Aug 19;19(8):e0308992. doi: 10.1371/journal.pone.0308992 (PMC11332943; doi:10.1371/journal.pone.0308992)
Supplement: S1 Table — (PDF) [file pone.0308992.s002.pdf]

General EPIC	Movement within the Age-Friendly Tab	Synopsis Tab	Screening Tab and Sub Tabs under the Screening Tab	Additional Notes/Time/Total Clicks	Questions/Things to follow up (grey out where unclear)
Start Visit			Start with Screening Tab in EPIC SmartSet (#1)	Start time 3:54	
	Age-Friendly Evaluation Tab (#2)				
	What Matters (#3)				
	Family togetherness (#4)				
	Health (#5)				
	Plan of care consistent to plan of patient (yes) (#6)				
Vital and History Tab (#7)					
Medication List (#8)					
Taking Lipitor (#9)					
Taking Motrin (#10)					
Taking Cozaar (#11)					

Taking Macrobid (#12)					
Taking Ceffexor-XR (#13)					
Mark as reviewed (#14)					
		Synopsis Tab (#15)			
		Beers/Non-beers medication Tab (#16)			
			Click on Screening Tab (#17)		
			Click Age-Friendly Tab (#18)		
	Click 4Ms to reactivate (#19)				
	Click yes to is the patient on any medication (#20)				
	According to synopsis any Beer – click yes (#21)				
	Actions to prescriptions - educate on risk of Beer criteria medication (#22)				
	Actions taken in relation to 4Ms – click yes (#23)				
	Re-clicked Age-Friendly				

	evaluation tba (#24)				
	Click Mentation above (25)				
			Mini cog assessme nt sub tab No 1 (2#6)		
			Banana, Sunrise, chair – I understa nd (#27)		
			Draw the clock – clicked normal (#28)		
			Repeat banana, sunrise, chair – click I understa nd (#29)		
			Banana – clicked No – (#30)		
			Sunrise - clicked yes (#31)		
			Chair – clicked no – (#32)		
			Close Tab (#33)		
			Subtab No 2 – Depressio		

			n screening (#34)		
			Do you feel less pleasure in doing things – clicked several days (#35)		
			Do you feel down or depressed or helpless – several days (#36)		
	Click Age-Friendly Evaluation Tab (#37)				
	Click Mentation above (#38)				
	Did you perform test for depression/delirium - yes (#39)				
	PHQ2-9 complete – yes (#40)				
	Minicog complete – yes (#41)				
	Was PHQ2-9/Minicog abnormal – No (#42)				

	Higher level of care referral – No (#43)				
	Actions taken - promote social engagement (#44)				
	4M brochure given – yes (#45)				
	Click Mobility above (#46)				
	Did you assess patient mobility – yes (#47)				
	Independently ambulatory – yes (#48)				
	Timed Get-Up-and-Go test done - yes (#49)				
	Clicked Normal (#50)				
	Encouraged to have daily mobility goal (#51)				
		Clicked Notes (#52)			
		Edit (#53)			
		Type MC 4MC and click to pull up note (#54)			
		Click on right side of EPIC – there's an arrow (#55)			
		Click documentation list (#56)			

		Click patient personal document (#57)			
		Type AF 4Ms brochure and scan into EPIC			
				Stop time 15:28<br>Total number of typing =2<br>Total number of clicks =57	
