# Supplementary material for: A human centered design approach to define and measure documentation quality using an EHR virtual simulation
Source: PLoS One. 2024 Aug 19;19(8):e0308992. doi: 10.1371/journal.pone.0308992 (PMC11332943; doi:10.1371/journal.pone.0308992)
Supplement: S2 Table — (PDF) [file pone.0308992.s003.pdf]

HPi:
67-year-old woman with a complaint of right-sided ear pain of a 3-day duration that comes and goes.
Patient should respond “No” to are you dizzy? And “No” to Is your hearing lessened since the right ear pain started? History of ear pain in the past with resolution...but cannot remember the details.

Mentation: Depression Screening
On PHQ2 you replied that Over the past 2 weeks you have been bothered by feeling down, depressed, or hopeless several days. Score of 1 on that second question, necessitates performing the full PHQ9. that forces the PhQ9?
Mentation: MiniCOG
She has a history of some memory issues and can only remember 1 word on the Mini-cog but can draw clock. Her score on Minicog is 3. 2 for the clock and 1 for getting one word correct. Normal score. This should be written down on Brochure for PCP to know.

What Matters:
What matters to her is her health.

Mobility:
She does no formal physical activity but tries to stay active and plays bingo once a week at local senior center. She has no gait abnormalities.

Her medications include:
Wellbutrin (For Anxiety and Depression). A SNRI which is a Beers med that could contribute to falls. She denies having any falls recently. Has been on Wellbutrin for 2 years.
Claritin (An antihistamine that should not cause anticholinergic effects like Benadryl would). She denies taking any Benadryl in addition nor having any anticholinergic effects like dry mouth, constipation, confusion.
Macrobid (For UTI Suppression). A Beers med that could cause peripheral Neuropathy. She denied numbness or tingling or extremities. Has been on this med for 1 year.
