# Supplementary material for: A human centered design approach to define and measure documentation quality using an EHR virtual simulation
Source: PLoS One. 2024 Aug 19;19(8):e0308992. doi: 10.1371/journal.pone.0308992 (PMC11332943; doi:10.1371/journal.pone.0308992)
Supplement: S3 Table — (PDF) [file pone.0308992.s004.pdf]

Please enter the following meds as HOME MEDS for our fake patient. Also see attached for the Fake Case Details. Enter these details as they come up in the visit.
The fake patient that you are going to talk to on the webex, has this same info to respond to you.

Wellbutrin (For Anxiety and Depression). A SNRI which is a Beers med that could contribute to falls.
When you ask her about this med, she will reply: she denies having any falls recently. Has been on Wellbutrin for 2 years.

Claritin (An antihistamine that should not cause anticholinergic effects like Benadryl would).
When you ask her about this med, she will reply she denies taking any Benadryl in addition nor having any anticholinergic effects like dry mouth, constipation, confusion.

Macrobid (For UTI Suppression). A Beers med that could cause peripheral Neuropathy.
When you ask her about this med, she will reply she denied numbness or tingling or extremities.
Has been on this med for 1 year.
