# Supplementary material for: A human centered design approach to define and measure documentation quality using an EHR virtual simulation
Source: PLoS One. 2024 Aug 19;19(8):e0308992. doi: 10.1371/journal.pone.0308992 (PMC11332943; doi:10.1371/journal.pone.0308992)
Supplement: S4 Table — (PDF) [file pone.0308992.s005.pdf]

Question	Strongly disagree	Disagree	Neutral	Agree	Strongly agree
I think that I would like to use this Age-Friendly 4Ms Screening Tab frequently.	☐	☐	☐	☐	☐
I found the Age-Friendly 4Ms Screening Tab unnecessarily complex.	☐	☐	☐	☐	☐
I thought the Age-Friendly 4Ms Screening Tab was easy to use.	☐	☐	☐	☐	☐
I think that I would need the support of a technical person to be able to use this Age-Friendly 4Ms Screening Tab.	☐	☐	☐	☐	☐
I found the various functions in this Age-Friendly 4Ms Screening Tab were well integrated.	☐	☐	☐	☐	☐
I thought there was too much inconsistency in this Age-Friendly 4Ms Screening Tab.	☐	☐	☐	☐	☐
I would imagine that most people would learn to use this Age-Friendly 4Ms Screening Tab.	☐	☐	☐	☐	☐
I found the Age-Friendly 4Ms Screening Tab very cumbersome to use.	☐	☐	☐	☐	☐
I felt very confident using the Age-Friendly 4Ms Screening Tab.	☐	☐	☐	☐	☐
I needed to learn a lot of things before I could get going with this Age-Friendly 4Ms Screening Tab.	☐	☐	☐	☐	☐
