# Supplementary material for: A human centered design approach to define and measure documentation quality using an EHR virtual simulation
Source: PLoS One. 2024 Aug 19;19(8):e0308992. doi: 10.1371/journal.pone.0308992 (PMC11332943; doi:10.1371/journal.pone.0308992)
Supplement: Attachment — Submitted filename: Response to Reviewers .docx [file pone.0308992.s006.docx]

July 24, 2024

Shadab Alam, Ph.D.

Academic Editor

PLOS ONE

RE: PLOS ONE Decision: Revision required [PONE-D-24-15723] - [EMID:294cc62d7fb41890]

Dear Dr. Alam,

We thank the reviewers for their insightful and constructive suggestions to improve the manuscript PONE-D-24-15723. We have attempted to address all of the reviewers’ comments and concerns to the best of our ability. Below is a point-by-point response to the reviewers. We have included detailed reviewer comments, for context and clarity. Additionally, comments have been added to the manuscript to highlight where we have addressed the feedback.

Per the recommendations of the editor, we deleted the funders acknowledgement in the text and pasting what we would like in the publication here “We are grateful for the support provided by The John A. Hartford Foundation <https://www.johnahartford.org/>, Author recipient: Mary A. Dolansky, Award Number: SPN00358. Based in New York City, the Foundation is a private, nonpartisan, national philanthropy dedicated to improving the care of older adults. For more than three decades, they have been leaders in the field of aging and health, focusing on creating age-friendly health systems, supporting family caregivers, and improving serious illness and end-of-life care. Their invaluable support has played a crucial role in the success of our project.”

Please let me know if you need anything further. Thank you again for consideration of our revised manuscript.

Sincerely,

Corresponding author

Megha Kalsy, PhD, MS

Data Scientist

Salt Lake City ORD VISN-19

U.S. Department of Veterans Affairs

Adjunct Assistant Professor

Frances Payne Bolton School of Nursing

Case Western Reserve University

megha.kalsy@utah.edu

megha.kalsy@va.gov

+1(440)985-8404 (cell)

**Itemized Response to Reviewer Comments**

Original Manuscript Title: A Human Centered Design Evaluation of Electronic Health Record Documentation Quality: A Case Study

**New Manuscript Title: A Human Centered Design Approach to Define and Measure Documentation Quality using an EHR Virtual Simulation**

**Ref.:  PONE-D-24-15723**

| **GENERAL JOURNAL REQUIREMENTS** | |
| --- | --- |
|  |  |
| **Comment** | **Addressed By** |
| 1) Please ensure that your manuscript meets PLOS ONE's style requirements, including those for file naming. | We have used the PLOS ONE file naming requirements. |
| 2) In the ethics statement in the Methods, you have specified that verbal consent was obtained. Please provide additional details regarding how this consent was documented and witnessed, and state whether this was approved by the IRB. | We have provided details about how this consent was documented and witnessed by 3 team members, and that this was approved by the Case Western Reserve University IRB.  Addressed on Page 10 |
| 3) Please remove any funding-related text from the manuscript and let us know how you would like to update your Funding Statement. | We have removed the funding-related statements from the manuscript and included them on the first page (second paragraph) of this rebuttal letter. |
| 4) Please include your full ethics statement in the ‘Methods’ section of your manuscript file. In your statement, please include the full name of the IRB or ethics committee who approved or waived your study, as well as whether or not you obtained informed written or verbal consent. If consent was waived for your study, please include this information in your statement as well. | We have included the full name of the IRB who approved our study. We also included information about the informed consent process.  Addressed on Page 9-10 |
| 5) Please review your reference list to ensure that it is complete and correct. | We have reviewed the reference list for accuracy.  We have added the following references:  Gopidasan B, 2022; Feldman J, 2023; Muylle KM, 2023; Bacchi S, 2023.  Addressed in References #7, #30, #31, #32. |
|  |  |
| **SPECIFIC REVIEWER COMMENTS** | |
|  |  |
| **Comment** | **Addressed By** |
| 1)    The researcher's contributions need to be better clarified | We are assuming that the reviewer means “aim/purpose” of the paper when stating “contribution”? We have changed the title to reflect the purpose listed in the abstract and discussion. The new title is “A Human Centered Design Approach to Define and Measure Documentation Quality using an EHR Virtual Simulation.” Briefly the contribution of the manuscript is to use a human centered design approach to define documentation and propose a method to measure documentation quality (timeliness, accuracy, user-centeredness, and efficiency) using a virtual simulated standardized patient visit via an EHR vendor platform by observing and recording documentation efforts. |
| 2) Since all the lines in Table 3 are units of measurement in seconds, there is no need to repeat them and just mention them in the title of the first column. | Table 3 was edited and the seconds were removed.  Addressed on Page 15 |
| 3) The researcher did not cover his research topic by including sufficient figures | We are assuming the reviewer is pointing to the HCD embedded in the research. We added narrative and a figure (Fig. 2) to cover this topic. Currently there are 3 figures within this paper.  Addressed on Page 6 |
| 4) The researcher did not provide sufficient comparisons to the works presented in this field | We added to the discussion additional comparisons to the work in this field.  Addressed on Page 17-20 |
| 5) The determinants presented by the researcher are very simple and require a fuller explanation along with using the results to create a clearer vision | We are assuming that the reviewer means dimensions of documentation quality? We added specifics to the description of the measurement in Table 1.  Addressed on Page 11 |
| 6) Inclusion of additional references published for the years 2023 and 2024 | We added updated references Gopidasan B, 2022. We included 3 updated references from 2023: Feldman J, 2023; Muylle KM, 2023; Bacchi S, 2023.  Addressed in References #7, #30, #31, #32. |
|  | |
| 1) The abstract is comprehensive but could be more concise. Consider summarizing the key points more succinctly. | We added the purpose statement to the abstract to capture the study’s essence.  Addressed on Page 2-3 |
| 2) Methods   The study design is appropriate, and the inclusion criteria for participants are well-defined.  Data collection methods are detailed and suitable for the research question.  The analysis methods are generally well-described. However, further clarification on specific statistical techniques used could enhance the understanding. | We added a section prior to results that summarizes the statistical techniques used.  Addressed on Page 13 |
| 3) Discussion   The discussion interprets the results effectively, relating them to existing literature and highlighting the implications for EHR documentation improvements.  The limitations of the study are acknowledged. The discussion could benefit from more detailed exploration of potential solutions to the identified issues. | Thank you for the feedback. We added potential solutions to each section in the discussion.  Addressed on Page 17-20 |
| 4) Language and Clarity   While the manuscript is generally clear, there are a few typographical and grammatical errors. For example:  In the abstract: "The data obtained from the assessment of documentation quality can be used in efforts to improve the documentation, user experience, and the quality of the data used in a Learning Healthcare System, with the goal of improving healthcare." Consider rephrasing for clarity. | Thank you for this edit. We reviewed the manuscript for typographical errors and changed the language in the abstract.  Abstract Addressed on Page 2-3. Typological errors addressed throughout the manuscript. |
| 5) In the Methods section: Ensure consistency in the use of terms such as "Age-Friendly 4Ms Screening Tab" and "AFHS 4Ms documentation." | Thank you for this feedback. We corrected line 224 from “AFHS” to Age-friendly to avoid confusion. We also noted inconsistency in our language with “screening tab and evaluation tab” and corrected this. We also included in the purpose statement what Age-Friendly 4Ms documentation is.  Addressed on Page 10 |
| 6) In the Discussion section: "This highlights the need for user documentation education to reduce erroneous time, particularly for categories with higher mean times." The phrase "user documentation education" could be clarified. | Clarified what is meant by documentation education.  Addressed on Page 19 |
| 7) Specific Errors   Inconsistent capitalization of "Nurse Practitioners" vs. "nurse practitioners." | Completed.  Addressed throughout the manuscript |
| 8) Occasional missing articles, e.g., "the" before "timeliness" in some sentences. | Completed  Addressed throughout the manuscript |
| 9) Minor punctuation errors, such as missing commas. | Thank you, reviewed and completed  Addressed throughout the manuscript |
| 10) Recommendations for Improvement   Abstract: Make it more concise, ensuring it captures the study's essence within the word limit. | We added the purpose statement to the abstract to capture the study’s essence.  Addressed on Page 2 |
| 11) Methods: Further clarify the data analysis process. | Thank you for this suggestion. We added a section before results to clarify the data analysis process. Addressed on Page 13 |
| 12) Discussion: Expand on potential solutions to the issues identified in the EHR documentation process. | Addressed on Page 17-20 |
